# CD73 expression in normal, hyperplastic, and neoplastic thyroid: a systematic evaluation revealing CD73 overexpression as a feature of papillary carcinomas

**DOI:** 10.1007/s00428-021-03100-x

**Published:** 2021-05-21

**Authors:** Inês Monteiro, Edoardo Missiaglia, Amedeo Sciarra, João Vasco Santos, Justine Bouilly, Pedro Romero, Christine Sempoux, Laurence de Leval

**Affiliations:** 1 Institute of Pathology, Department of Laboratory Medicine and Pathology, Lausanne University Hospital and University of Lausanne, Rue du Bugnon 25, CH-1011 Lausanne, Switzerland; 2 Department of Histopathology, Central Institute, Valais Hospital, Sion, Switzerland; 3 MEDCIDS - Department of Community Medicine, Information and Health Decision Sciences, Faculty of Medicine, University of Porto, Porto, Portugal; 4 CINTESIS - Centre for Health Technology and Services Research, Porto, Portugal; 5 Public Health Unit, ACES Grande Porto VIII - Espinho/Gaia, ARS Norte, Espinho, Portugal; 6 Department of Fundamental Oncology, University of Lausanne, Lausanne, Vaud Switzerland

**Keywords:** CD73, Immunohistochemistry, Thyroid adenoma, Thyroid carcinoma, Papillary thyroid carcinoma

## Abstract

CD73 converts AMP to adenosine, an immunosuppressive metabolite that promotes tumorigenesis. This study presents a systematic evaluation of CD73 expression in benign, hyperplastic, and neoplastic thyroid. CD73 expression was assessed by immunohistochemistry in 142 thyroid samples. CD73 was expressed in normal thyroid (3/6) and goiter (5/6), with an apical pattern and mild intensity. Apical and mild CD73 expression was also present in oncocytic cell adenomas/carcinomas (9/10; 5/8) and in follicular adenomas/carcinomas (12/18; 23/27). In contrast, papillary thyroid carcinomas featured extensive and intense CD73 staining (49/50) (vs. normal thyroid/goiter, *p* < 0.001). Seven of nine anaplastic carcinomas were CD73-positive with heterogeneous extensiveness of staining. Medullary and poorly differentiated carcinomas were mostly CD73-negative (1/6; 2/2). These results were corroborated by *NT5E* mRNA profiling. Papillary carcinomas feature enhanced CD73 protein and mRNA expression with distinct and intense staining, more pronounced in the invasive fronts of the tumors.

**Supplementary Information:**

The online version contains supplementary material available at 10.1007/s00428-021-03100-x.

## Introduction

CD73, encoded by the *NT5E* gene, is an ectonucleotidase which produces adenosine [1]. Adenosine is often present in the tumor microenvironment and can lead to immunosuppression and epithelial-mesenchymal transition of tumor cells, correlating with poor prognosis in several tumor types [1, 2]. Also, higher levels of CD73 correlate with refractoriness to cancer treatments [1, 3].

New evidence pointing the possibility to restore immune response via CD73 blockade has triggered a characterization of CD73 expression in human cancers [4].

Few data are available on CD73 expression in thyroid cancer. Increased CD73 expression and enzymatic activity in papillary thyroid carcinoma (PTC, *n* = 3) vs. normal thyroid, goiter, and follicular adenoma (*n* = 9) was reported [5]. Another report described increased *NT5E* mRNA and protein levels in PTC vs. normal thyroid cell lines [6]. The same group showed that the PTC/adjacent paired expression ratio of *NT5E* mRNA was associated with metastatic lymph nodes and tumor size and that all PTC (*n* = 29) were CD73-positive by immunohistochemistry (IHC) [7].

In the present study, we examine CD73 expression using both IHC and mRNA profiling in a large series of normal, hyperplastic, and neoplastic thyroid. This study aims to complete and nuance the early data regarding CD73 expression in thyroid neoplasms.

## Methods

Formalin-fixed paraffin-embedded surgical thyroid specimens were retrieved from the archives of the Institute of Pathology of the Lausanne University Hospital (1995–2019). Cases with the desired coded diagnosis, patient consent, and available material were chosen. Six cases each of “normal thyroid” and “goiter” were chosen. For the remaining diagnostic categories, a total of 130 eligible cases were found. One block per patient was selected. All diagnoses were confirmed.

Immunohistochemistry, RNA profiling using HTG EdgeSeq Oncology Biomarker Panel, and quantification of tumor-infiltrating mononuclear cells (TIMC) are described in Supplementary Methods.

## Results

### CD73 immunohistochemical expression

Results are summarized in Table 1.

**Table 1:** CD73 expression in 142 benign, hyperplastic and neoplastic surgical thyroid samples

Lesion (*n* = 142)	Staining distribution, pattern	CD73+ N/total (%)	CD73 intensity	H-scoreMedian (min-max)
Normal thyroid (*n* = 6)	Follicles, apical	3/6 (50)	Mild (3/3)	20 (0–75)
Goiter (*n* = 6)	Follicles, apical	5/6 (83)	Mild (4/5) Moderate (1/5)	7.5 (0–80)
Follicular adenoma (*n* = 18)	Follicles, apical	12/18 (67)	Mild (11/12) Moderate (1/12)	21.25 (0–120)
Oncocytic cell adenoma (*n* = 10)	Trabecular, negative/follicles, apical	9/10 (90)	Mild (7/9) Moderate (1/9) Strong (1/9)	11.25 (0–50)
Follicular carcinoma (*n* = 27)	Follicles, apical	23/27 (85)	Mild (23/23)	22.5 (0–90)
Primary (*n* = 21)		18/21 (86)	Mild (18/18)	20 (0–90)
Metastatic (*n* = 6)		5/6 (83)	Mild (5/5)	23.75 (0-40)
Oncocytic cell carcinoma (*n* = 8)	Trabecular, negative/follicles, apical	5/8 (63)	Mild (5/5)	17.5 (0–105)
Papillary carcinoma (*n* = 50)	Accentuated in papillae and in advancing edge. Membranous and cytoplasmic staining	49/50 (98)	Mild (17/49) Moderate (22/49) Strong (10/49)	120 (0–285)
Primary (*n* = 39)		38/39 (97)	Mild (10/38) Moderate (22/38) Strong (7/38)	120 (7.5–285)
Metastatic (*n* = 11)		11/11 (100)	Mild (8/11) Strong (3/11)	60 (7.5–225)
Poorly differentiated carcinoma (*n* = 2)	Mostly negative. Faint cytoplasmic staining	2/2 (100)	Mild (2/2)	8.75 (7.5–10)
Anaplastic carcinoma (*n* = 9)	Membranous and cytoplasmic staining	7/9 (78)	Mild (1/7) Moderate (3/7) Strong (3/7)	50 (0–237.5)
Medullary carcinoma (*n* = 6)	Mostly negative	1/6 (17)	Mild (1/1)	0 (0–5)

*Mild staining* intensity of 1 or 1.5, *moderate staining* intensity of 2, *strong staining* intensity of 2.5 or 3

**Fig. 1:**
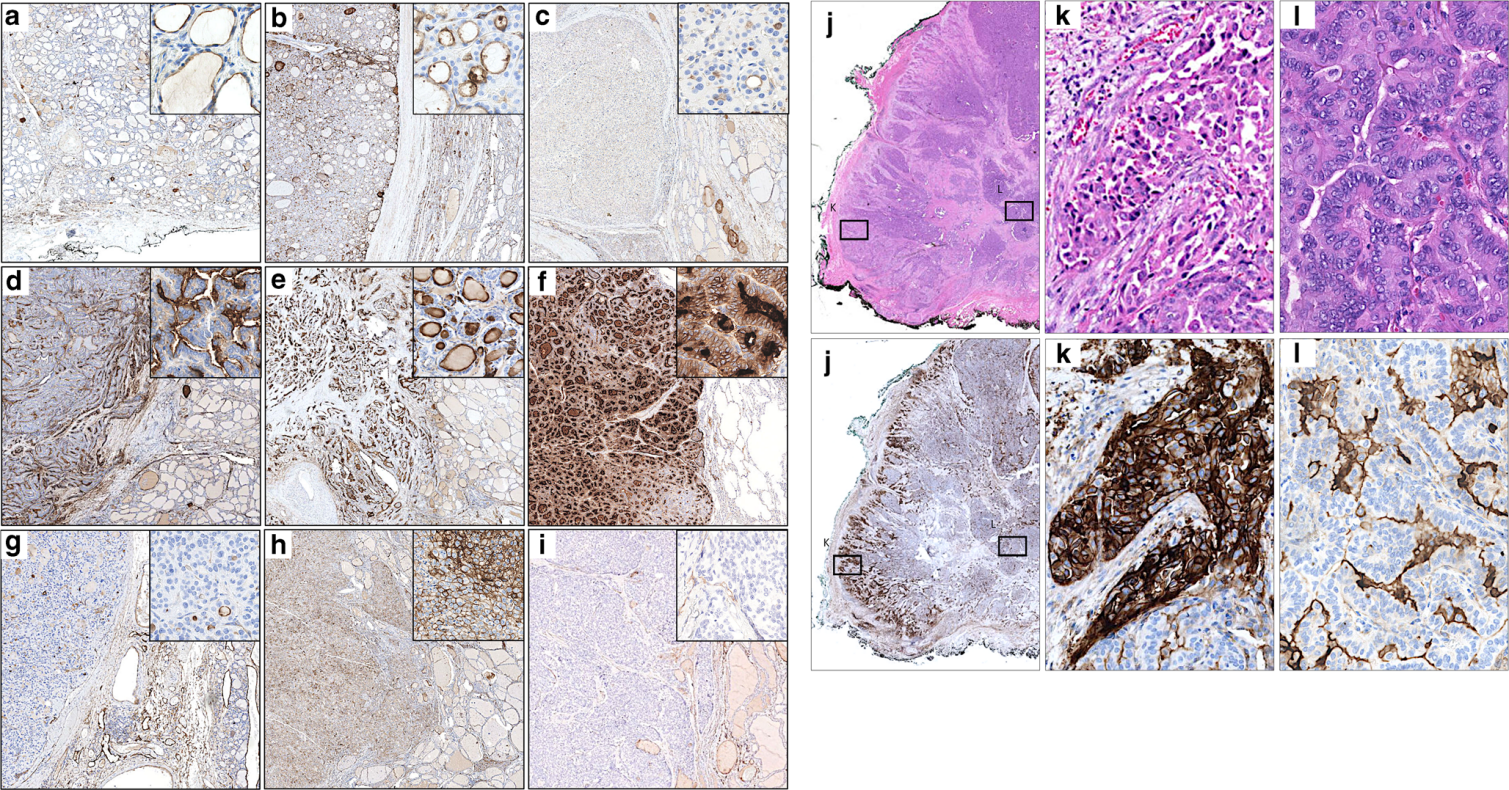
CD73 immunohistochemistry in benign and neoplastic thyroid. **a** Normal thyroid. CD73 is expressed in follicles, at the apical pole of the membrane of thyrocytes (inset), with mild intensity. **b** Follicular carcinoma. CD73 expression in the carcinoma (left) is apical (inset) and similar to that in non-neoplastic thyroid (right). **c** Oncocytic carcinoma. CD73 expression is limited to areas with follicular architecture (inset). **d** Papillary carcinoma. CD73 is expressed in the apical pole of the papillae with strong intensity (inset). **e** Papillary carcinoma with follicular morphology. The follicles also show strong CD73 expression (inset). **f** Lung metastasis of papillary carcinoma with predominantly follicular morphology. Follicles showing strong apical staining, extension to the basolateral membrane and cytoplasmic staining (inset). **g** Poorly differentiated carcinoma. Poorly differentiated insular/trabecular areas are CD73-negative, while follicular areas present some CD73 positivity (inset). **h** Anaplastic carcinoma. Diffuse CD73 expression of variable intensity. **i** Medullary carcinoma. Negative CD73 staining. **j–l** Hematoxylin and eosin (upper panels) and corresponding CD73 IHC (lower panels) at various magnifications of an invasive front of a papillary thyroid carcinoma; **j** panoramic view of a papillary thyroid carcinoma with a stronger CD73 staining along the invasion front (left edge) compared to the central portion of the lesion; **k** (inset of (**j**), rotated) strong and complete membrane and cytoplasmic staining of the invasive front; **l** (inset of (**j**), rotated) less intense apical membrane staining of the central part of the lesion. Images **a–i** and respective insets were taken at an original magnification of × 2 and × 18, respectively. Images **j–l** were taken at an original magnification of × 0.23, × 6.40, and × 6.40, respectively

Most specimens of normal thyroid and goiter had detectable CD73 expression, characterized by an apical staining of mild intensity in follicular thyrocytes (3/6, 50%, median H-score 20; 5/6, 83%, median H-score 7.5, respectively) (Fig. 1a). This staining was mostly present in the membrane and rarely in the cytoplasm.

Most follicular adenomas presented CD73 staining (12/18, 67%, median H-score 21.25). Among follicular carcinoma samples, 21 were retrieved from primary and 6 from metastatic lesions (3 subcutaneous tissue metastases, 2 lymph node metastases, and 1 rib metastasis). Most follicular carcinomas presented CD73 staining (23/27, 85%, median H-score 22.5) (Fig. 1b). In these entities, CD73 staining distribution, pattern, and intensity were mostly similar to those of the adjacent non-neoplastic thyroid (normal thyroid/goiter vs. follicular adenomas/carcinomas, *p* = 0.414).

In oncocytic adenomas and carcinomas, CD73 was not expressed by cells arranged in a trabecular pattern; nonetheless, follicular areas presented a mild apical staining (9/10, 90%, median H-score 11.25; 5/8, 63%, median H-score 17.5, respectively) (Fig. 1c). Only one case of oncocytic adenoma presented foci of tumor cells staining with strong intensity. The H-scores of oncocytic tumors did not significantly differ from those of normal thyroid and goiter (*p* = 0.781).

**Fig. 2:**
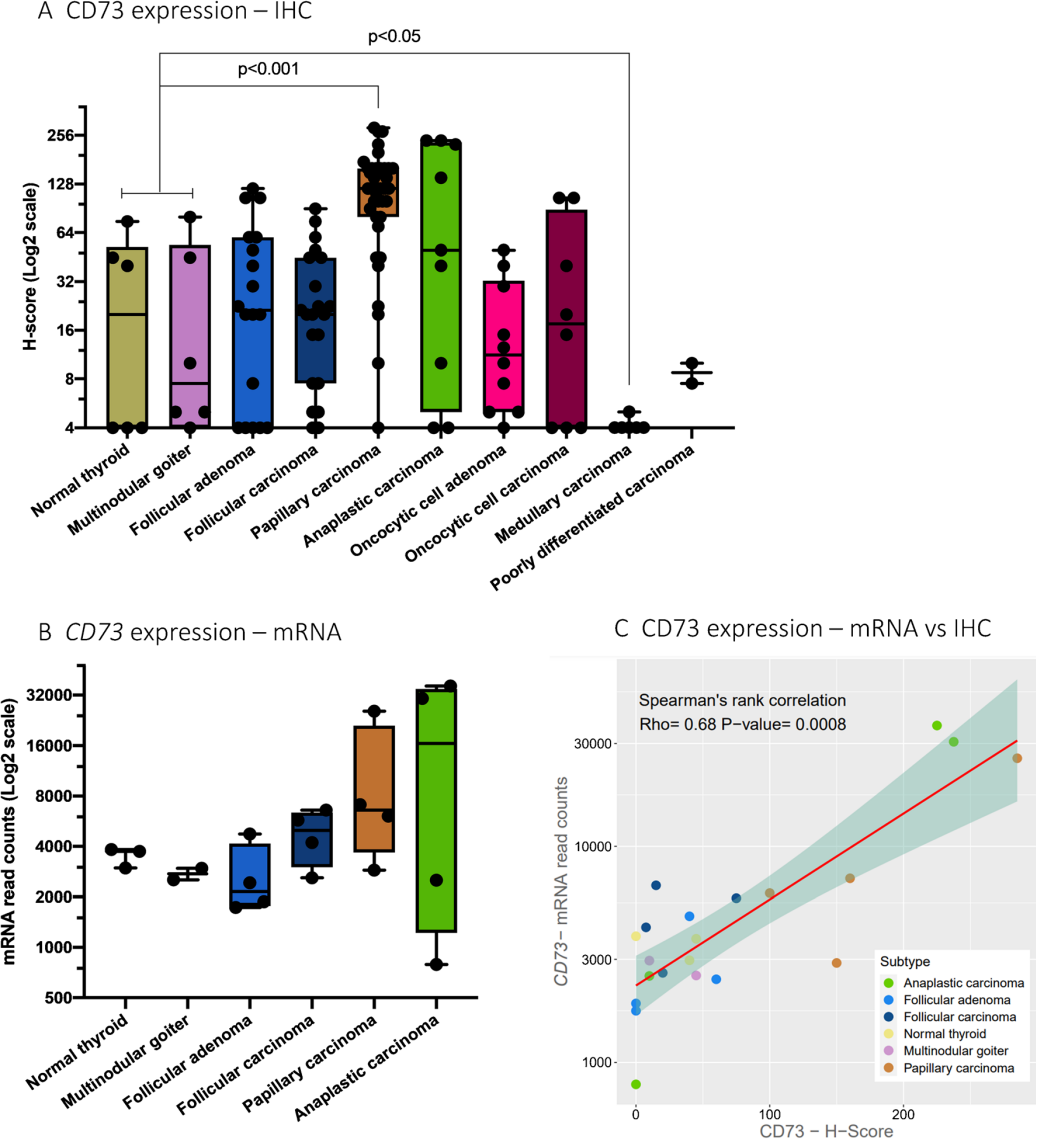
CD73 expression in benign, hyperplastic and neoplastic thyroid. **a** Summary of CD73 protein expression assessed by immunohistochemistry (IHC) expressed as H-scores in normal, hyperplastic and neoplastic thyroid (*n* = 142). *p* Values obtained using a Mann-Whitney *U* test; **b** *NT5E* mRNA expression in 21 samples analyzed with. HTG oncology immune panel; **c** correlation between CD73 IHC H scores and *NT5E* mRNA read counts in 21 samples analyzed with. HTG oncology immune panel

The vast majority of PTC either primary (*n* = 39) or metastatic (*n* = 11; involving lymph nodes (7), soft tissues (3), and lung (1)) expressed CD73 (49/50, 98%, median H-score 120) (Fig. 1d–f). Most primary PTC showed extension of CD73 expression to the basolateral membrane (43/50, 86%) and, occasionally, cytoplasmic staining. Areas with moderate/strong staining intensity were observed in 64% (32/50) of cases. The H-score of PTC was significantly higher compared to normal thyroid/goiter (*p* < 0.001) (Fig. 2a).

Interestingly, PTC with follicular morphology (*n* = 17) presented a staining pattern similar to that of classical PTC (staining extending to the basolateral membrane in 11/17 cases, of moderate/strong intensity in 12/17 cases) (Fig. 1e–f).

Remarkably, all cases of primary PTC with an invasive front (*n* = 23) distinctly comprised a higher proportion of tumor cells staining and/or higher intensity of staining in the invasive front, in comparison to the rest of the tumor (median H-score of the invasive fronts = 225, median global H-score of the respective tumors = 140, *p* < 0.001) (Fig. 1j–l).

Two cases of poorly differentiated carcinomas expressed CD73 with mild apical staining (2/2, 100%, median H-score 8.75) but only in small areas where cells were organized in follicles (Fig. 1g).

The majority of anaplastic carcinomas were CD73-positive (7/9, 78%, median H-score 50), presenting membrane and cytoplasmic staining (Fig. 1h). Inter- and intralesional staining was heterogeneous in terms of percentage of CD73-positive cells and staining intensity. This heterogeneity was reflected by an H-score of the positive cases (*n* = 7) ranging from 10 to 237.5 (Fig. 2a). The H-scores of anaplastic carcinomas did not significantly differ from those of normal thyroid and goiter (*p* = 0.113).

Finally, most medullary carcinomas were CD73-negative (Fig. 1i) as was also the case with hyperplastic parafollicular C-cells identified in the adjacent thyroid parenchyma (confirmed with calcitonin staining) in one case of multiple endocrine neoplasia. Only one medullary carcinoma contained a small proportion (5%) of tumor cells with faint CD73 staining (1/6, 17%, median H-score 0). The H-score of medullary carcinomas was significantly lower compared to normal thyroid/goiter (*p* = 0.035).

### *CD73 (NT5E)* mRNA expression

mRNA counts were higher in papillary carcinoma (*n* = 4, median read count 6591) than in normal thyroid (*n* = 3, median read count 3736) and multinodular goiter (*n* = 2, median read count 2742) (Fig. 2a–b). Regarding anaplastic carcinomas (*n* = 4, median read count 16532), mRNA counts were highly heterogeneous. Overall, we found that CD73 IHC scores correlated significantly with *NT5E* mRNA expression (Spearman’s rank correlation *p* = 0.0008) (Fig. 2c).

### Tumor-infiltrating immune cells

**Supplementary Fig. S1:**
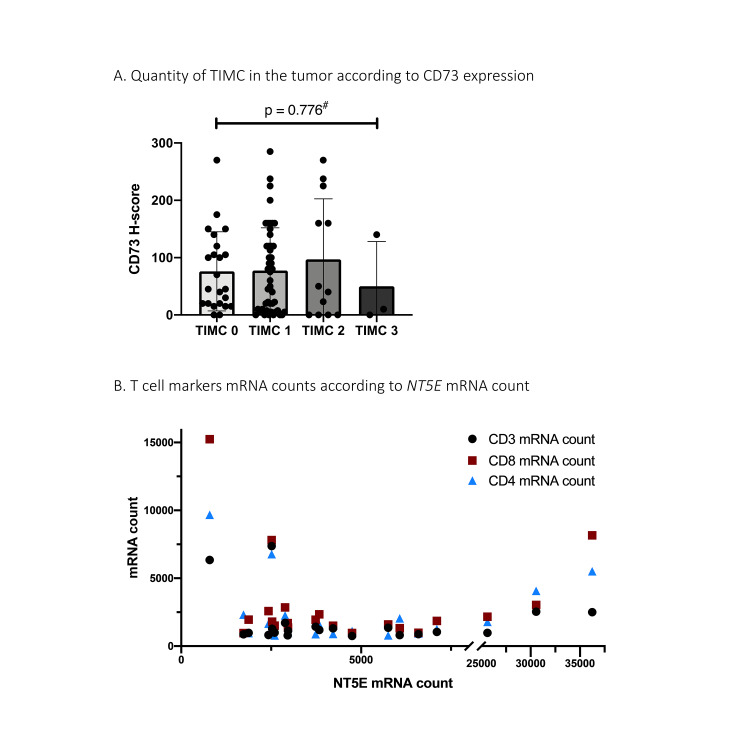
CD73 expression and tumor-infiltrating immune cells. A. CD73 expression (H-score) in the tumor cells of primary thyroid carcinomas (n = 85) per quantity of tumor infiltrating mononuclear cells (TIMC) present in the tumor, classified semi-quantitatively in four groups: none (0), scarce (1), moderate (2), abundant (3); B. *CD3*, *CD8* and *CD4* mRNA counts according to the mRNA counts of *NT5E* (n = 21). # one-way ANOVA. (PNG 297 kb)

Adenosine leads to immunosuppression by several mechanisms, namely by hampering immune effector cell functions and contributing to T cell exhaustion [8]. First, we seeked to characterize the density of TIMC in the primary malignant lesions (*n* = 85) and the eventual correlation with CD73 expression. Most lesions presented no or few TIMC. Only anaplastic carcinomas presented mostly a moderate or abundant quantity of TIMC. The abundance of TIMC did not correlate with CD73 expression in the tumor cells (one-way ANOVA *p* = 0.776) (Supplementary Fig. S1a).

In addition, in the 21 samples analyzed by HTG EdgeSeq, *CD3*, *CD8,* and *CD4* mRNA read counts did not correlate with *NT5E* mRNA read counts (Supplementary Table S1, Supplementary Fig. S1b). Further, no correlation between *NT5E* and *CTLA4*/CTLA4, *CD274*/PD-L1, and *PDCD1*/PD-1 mRNA read counts was found (Supplementary Table S1).

## Discussion

In this report, we evaluated CD73 expression in a series accounting for most thyroid lesions, using an immunohistochemistry assay corroborated by an mRNA analysis in a subset of cases.

Strikingly, we observed that PTC showed enhanced CD73 expression beyond the apical membrane, into the basolateral membrane and the cytoplasm, and that this phenomenon was even more prominent in the invasive front. This might reflect a functional role for CD73 in PTC invasiveness. Indeed, a recent study demonstrated that CD73 inhibition suppressed PTC cell migration in vitro and that CD73 expression was associated with lymph node metastasis, suggesting that CD73 is involved in the dissemination of PTC [9].

This extended pattern of staining was preserved in PTC with follicular morphology. This feature suggests that CD73 IHC could be of use in diagnostic practice to help distinguish follicular-patterned lesions which may pose the differential diagnosis between follicular versus papillary carcinoma.

The theory that anaplastic carcinomas usually develop from dedifferentiation of differentiated thyroid carcinomas is generally accepted [10]. Given the heterogeneity of CD73 expression in these carcinomas, we conjecture that tumors with higher H-scores could potentially have developed from PTC, while negative or faintly positive tumors could have developed from follicular carcinomas.

CD73 overexpression seen in papillary and anaplastic tumors might relate to the tumor microenvironment. It has been reported that mesenchymal stromal cells increase CD73 expression in tumor cells through TGF-β1 production [11]. Interestingly, in the 21 samples analyzed by HTG EdgeSeq, *NT5E* and *TGF-β1* mRNA were positively correlated (Spearman’s rank correlation *p* = 0.04) (data not shown).

Most medullary carcinomas and non-neoplastic parafollicular C-cells were CD73-negative, suggesting an absent CD73 expression in normal and neoplastic thyroid endocrine cells. A similar observation was made in our previous study regarding pancreatic neuroendocrine tumors [12].

**Supplementary Fig. S2:**
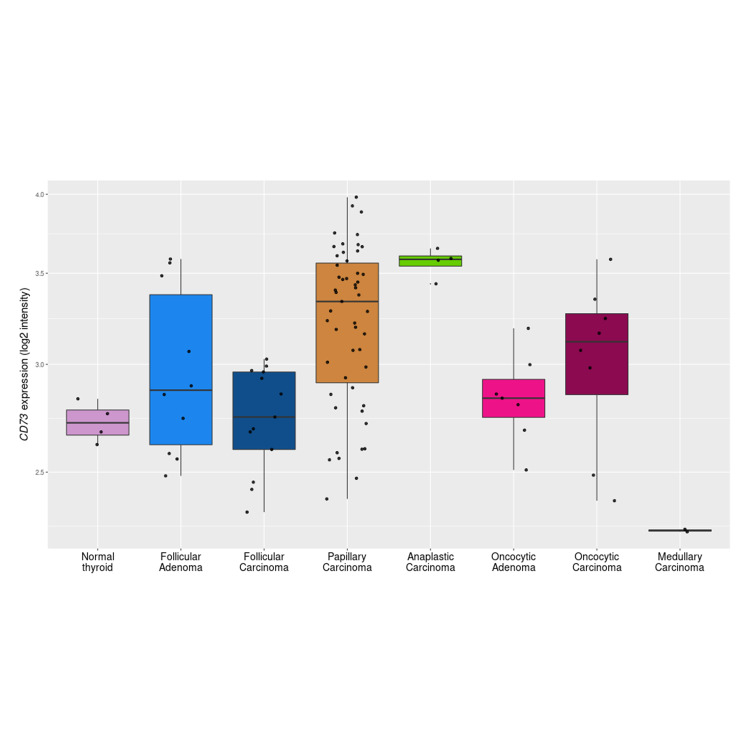
*CD73* (*NT5E*) mRNA expression in normal and neoplastic thyroid (n = 99). CD73 expression data were obtained from the GSE27155 set (GEO database) and derived from Affimetrix U133A arrays. Normalized data were retrieved and analyzed using GEO2R and ggplot2 packages and other functions in R software (v.3.6). (PNG 305 kb)

Our results regarding CD73 expression at the protein and mRNA levels are corroborated by the *NT5E* gene analysis retrieved from the Gene Expression Omnibus (GEO) database (GSE27155 set) [13] (Supplementary information Fig. S2).

No relationship between CD73 expression in the primary tumor and either the density of TIMC or the mRNA expression of T cell markers was found. In accordance with our findings, a recent study found no correlation between *CD73* and *CD8* transcript levels. However, a positive correlation between the expression of *CTLA4*, *CD274, PDCD1*, and *CD73* transcript levels was reported [9]. The number and distribution of tumor-infiltrating immune cells are determined by several factors, namely, the neovascular permeability, the gradients of various cytokines, and the composition of the extracellular matrix [14, 15]. Adenosine is likely to act locally, impairing immune cells in a paracrine fashion. Still, functional studies are needed to characterize the effect of CD73 expression in tumor-infiltrating immune cells.

In conclusion, CD73 is highly expressed in PTC, presenting a distinct IHC pattern. The high CD73 expression observed in PTC encourages to further explore the use of therapies targeting the CD73-adenosine pathway in this entity.

## Supporting information

ESM 1 (DOCX 21 kb) High resolution image (TIFF 2960 kb) High resolution image (TIF 7578 kb) ESM 4 (DOCX 13 kb)
