# Supplementary material for: CD73 expression in normal, hyperplastic, and neoplastic thyroid: a systematic evaluation revealing CD73 overexpression as a feature of papillary carcinomas
Source: Virchows Arch. 2021 May 21;479(1):209–14. doi: 10.1007/s00428-021-03100-x (PMC8298324; doi:10.1007/s00428-021-03100-x)
Supplement: ESM 1 — (DOCX 21 kb) [file 428_2021_3100_MOESM1_ESM.docx]

**Supplementary Table 1.** mRNA expression of *NT5E* (*CD73*), T cell markers and T cell checkpoint inhibitors in benign, hyperplastic and neoplastic thyroid samples

NA: not applicable; TIMC: tumor-infiltrating mononuclear cells.

| **Diagnostic categories** (n=21) | **CD73**  **H-score**  **(0-300)** | ***NT5E* (CD73) mRNA count** | **Quantification of TIMC**  **(0, 1, 2, 3)** | **T cell markers** | | | **T cell checkpoint inhibitors** | | |  |
| --- | --- | --- | --- | --- | --- | --- | --- | --- | --- | --- |
|  |  |  |  | ***CD3* mRNA count** | ***CD8* mRNA count** | ***CD4* mRNA count** | ***PDCD1* (PD-1) mRNA count** | ***CD274* (PD-L1) mRNA count** | ***CTLA4* mRNA count** | |
| **Normal thyroid**  (n=3) | 0 | 3839 | NA | 1186 | 2339 | 1578 | 1005 | 1244 | 585 | |
|  | 40 | 2971 | NA | 1123 | 1362 | 1021 | 1034 | 1278 | 470 | |
|  | 45 | 3736 | NA | 1435 | 1939 | 877 | 1204 | 1860 | 890 | |
| **Multinodular goiter** (n=2) | 10 | 2960 | NA | 790 | 1698 | 1025 | 509 | 952 | 431 | |
|  | 45 | 2526 | NA | 1280 | 1799 | 1636 | 406 | 1269 | 530 | |
| **Follicular adenoma**  (n=4) | 0 | 1732 | 0 | 867 | 959 | 2320 | 561 | 760 | 370 | |
|  | 0 | 1873 | 0 | 973 | 1949 | 957 | 843 | 1104 | 489 | |
|  | 40 | 4751 | 0 | 749 | 966 | 1108 | 639 | 2673 | 423 | |
|  | 60 | 2425 | 0 | 820 | 2582 | 1648 | 735 | 1403 | 507 | |
| **Follicular carcinoma** (n=4) | 7.5 | 4222 | 1 | 1309 | 1504 | 895 | 1454 | 1752 | 867 | |
|  | 15 | 6596 | 0 | 883 | 982 | 935 | 728 | 1233 | 409 | |
|  | 20 | 2597 | 0 | 990 | 1510 | 784 | 1151 | 1318 | 742 | |
|  | 75 | 5752 | 1 | 1353 | 1597 | 790 | 1112 | 1730 | 823 | |
| **Papillary carcinoma** (n=4) | 100 | 6075 | 1 | 812 | 1315 | 2049 | 718 | 1571 | 469 | |
|  | 150 | 2887 | 1 | 1713 | 2853 | 2259 | 1053 | 2708 | 856 | |
|  | 160 | 7108 | 1 | 1043 | 1849 | 1257 | 753 | 1747 | 600 | |
|  | 285 | 25625 | 1 | 979 | 2171 | 1782 | 1028 | 1340 | 584 | |
| **Anaplastic carcinoma** (n=4) | 0 | 792 | 3 | 6344 | 15246 | 9669 | 2617 | 2800 | 1699 | |
|  | 10 | 2513 | 3 | 7368 | 7804 | 6773 | 2050 | 2730 | 2995 | |
|  | 225 | 36238 | 2 | 2502 | 8161 | 5510 | 930 | 12931 | 1066 | |
|  | 237.5 | 30552 | 2 | 2531 | 3031 | 4067 | 1077 | 1540 | 991 | |
