# Supplementary material for: CD73 expression in normal, hyperplastic, and neoplastic thyroid: a systematic evaluation revealing CD73 overexpression as a feature of papillary carcinomas
Source: Virchows Arch. 2021 May 21;479(1):209–14. doi: 10.1007/s00428-021-03100-x (PMC8298324; doi:10.1007/s00428-021-03100-x)
Supplement: ESM 4 — (DOCX 13 kb) [file 428_2021_3100_MOESM4_ESM.docx]

**Supplementary Methods**

Immunohistochemistry: IHC was performed using a CD73-specific antibody (D7F9A, rabbit monoclonal, #13160, Cell Signaling), using the Ventana BenchMark automated stainer. Briefly, deparaffinized slides were pre-treated with CC1 and then incubated with the anti-CD73 antibody. The Ultraview DAB detection kit (ref. 760–500) was used.

We qualified the cellular staining distribution (membrane, cytoplasmic or both). For each specimen, we evaluated the percentage of CD73-positive cells (0-100%) and the staining intensity (negative=0, mild=1 or 1.5, moderate=2, strong=2.5 or 3). An H-score was calculated as the percentage of tumors cells staining multiplied by the intensity of staining (score ranging from 0 to 300). Comparisons of H-score were performed using Mann Whitney U tests. All cases were scored by IM or AS and reviewed with LDL or CS. Staining in endothelium and stroma was used as positive internal control.

RNA profiling using HTG EdgeSeq Oncology Biomarker Panel: 21 of the 142 FFPE specimens were selected for RNA profiling. Representative cases of the main diagnostic categories were randomly selected (3 normal thyroids, 2 multinodular goiters, 4 follicular adenomas, 4 follicular carcinomas, 4 papillary carcinomas and 4 anaplastic carcinomas). HTG EdgeSeq libraries were generated according to the HTG EdgeSeq workflow. The area of interest for molecular profiling (minimal area 6mm^2^ with a 5µm thickness) was selected using adjacent HE stained slides, collected by scraping and put in HTG lysis buffer and proteinase K for RNA extraction. Libraries were prepared with the HTG EdgeSeq system using the Oncology Biomarker Panel kit, followed by sample barcoding using PCR-based amplification. Final libraries were cleaned up with AMPure beads, quantified by qPCR with KAPA Library Quantification Kits and pooled according to the manufacturer’s protocol. Sequencing was performed by NextSeq (Illumina) and FASTQ files were analyzed by HTG software. Raw counts were imported in R software (v.3.6.0) and normalized by size factor using DESeq2 package (v.1.26.0). Correlation between normalized RNA counts and H-score was assessed using non-parametric Spearman’s rank correlation test in R. Post-sequencing quality controls were used to detect and exclude samples with either degraded RNA, insufficient read depth or minimal expression variability.

Quantification of tumor-infiltrating mononuclear cells (TIMC): Using Hematoxylin & Eosin (HE) slides, the quantity of TIMC in primary malignant lesions was reported semi-quantitatively in four groups (none (0), scarce (1), moderate (2), abundant (3)). TIMC were considered if they were intratumoral or peritumoral in case of capsular invasion. TIMC surrounding areas of intact capsule were not considered. For each of the diagnostic categories, the H-score of the tumors was compared between TIMC groups using a Kruskal-Wallis test.
